# Five ways to get a grip on evaluating and improving educational continuity in health professions education programs

**DOI:** 10.36834/cmej.69228

**Published:** 2020-09-23

**Authors:** Ann S. Lee, Shelley Ross

**Affiliations:** 1 University of Alberta, Alberta, Canada

## Abstract

Presence of educational continuity is essential for progressive development of competence. Educational continuity appears to be a simple concept, but in practice, it is challenging to implement and evaluate because of its multifaceted nature. In this Black Ice article, we present some practical tips to help avoid misunderstandings and irregularities in implementation for those involved in evaluating and improving educational continuity in health professions education programs.

## Abstract (fr)

La continuité pédagogique est primordiale pour assurer le développement progressif des compétences. Elle semble être un concept simple, mais, en pratique, elle est difficile à implanter et évaluer en raison de ses multiples facettes. Dans cet article, nous offrons des trucs pratiques aux responsables de l’évaluation et de l’amélioration de la continuité pédagogique dans les programmes de formation des professions de la santé, afin d’aider à éviter les malentendus et les irrégularités dans l’implantation de ce concept,

## Introduction

Educational continuity, or intentional connections between learning experiences, is increasingly recognized as essential to health professions education. This need for educational continuity is even more critical now as, around the world, we are in a time of major reform in health professions education heading towards competency-based medical education (CBME).^1-3^ One of the greatest challenges faced by health professions education program designers and educators is how to avoid the pitfalls of multiple discrete learning experiences fragmented by blocks of curricular time, punctuated by episodic patient or client (in the case of such health professions as veterinary medicine) care experiences, and divided among numerous teachers. This type of disjointed learning can be detrimental to retention and appropriate application of knowledge, as it may impede progressive development of competence.^3^

Many of the challenges around designing for educational continuity stem from the multifaceted nature of this construct. There is continuity of care (coherence and connection between a patient and provider^4^), continuity of curriculum (integration between foundational knowledge and clinical skills^5^), and continuity of supervision (relationship between the learner and educator^5^), all of which are essential (Table 1).^3^ However, implementing and evaluating these elements of a health professions training program is difficult due to the fact that while all of these concepts involve continuity, each is affected by different dimensions of context and curriculum, and each requires differences in process and approach (Table 1).^4-9^ Misunderstandings can arise when program designers, administrators, clinical teachers, and learners are conceptualizing different constructs of continuity, and are basing their assumptions about need for and importance of continuity on those internal definitions, which may differ from those held by others. These misunderstandings become even more of a barrier when attempting to carry out program evaluation for educational continuity.

We present here some practical tips and guidelines to help avoid the “black ice”, or misunderstandings and irregularities in implementation, for those involved in evaluating and improving educational continuity in health professions education programs.

***1. Focus on a specific dimension of educational continuity for each program evaluation project***

A good first step in program evaluation is to define the problem.^10^ In the case of educational continuity, this step is crucial, given the multiple dimensions of educational continuity. The three broad categories of educational continuity, “continuity of care”, “continuity of curriculum” and “continuity of supervision”, are all essential for the development of competence, albeit in different ways (Table 1). The ways in which each are implemented will appear quite different, so planning approaches to program evaluation, and the data that will need to be collected, will also differ.

**Table 1:** Definitions of educational continuity from the health professions education literature.

Construct	Definition
**Continuity of care**	“the degree to which a series of discrete healthcare events is experienced as coherent and connected and consistent with the patient’s medical needs and personal context.”^4^
**Subtypes^4,6^** Informational Management Relational/ Interpersonal	“The use of information on past events and personal circumstances to make current care appropriate for each individual.”^4^ “A consistent and coherent approach to the management of a health condition that is responsive to a patient’s changing needs.”^4^ “An ongoing therapeutic relationship between a patient and one or more providers.”^4^
**Dimensions^6-9^** Longitudinal Family Geographic Interdisciplinary Community Health care team	Care provided over time Care provided to family members Care provided in different settings Care as patients are followed across disciplines Care provided to members of the a community Care provided by a health care team
**Continuity of curriculum**	“the vertical integration between basic sciences and clinical medicine as well as horizontal integration across disciplines”^5^
**Continuity of supervision**	“the relationship between learner and teacher over time”^5^

Continuity of care is usually understood to be about the patient or client experience, but it is also crucial to the development of a learner’s clinical competence. Specifically, it is interpersonal continuity that is associated with a more complete educational experience for medical students and residents for the development of skills in interacting with patients over time and in managing chronic conditions.^11,12^ When a program evaluation project specifies continuity of care as the target, such elements as opportunities for developing relationships with patients over time and throughout an illness experience become the focus for evaluation and improvement.

Continuity of curriculum and continuity of supervision (Table 1) will each require a different focus in program evaluation, and further need for defining the problem depending on context (classroom teaching or clinical setting). Continuity of curriculum program evaluation projects will look at the vertical integration between basic sciences and clinical medicine as well as horizontal integration across disciplines.^4^ Measurement will focus on meaningful and relevant connections between static knowledge and application of that knowledge in clinical contexts. Continuity of supervision program evaluation projects will focus on how often learners are with the same supervisor(s) and/or in the same teaching units with the same team(s) as well as whether learners and supervisors feel that opportunities occur for development of effective educational alliances.^13^

***2. Collect both quantitative and qualitative data on educational continuity***

Once you have defined your target for your educational continuity program evaluation project, it is important to next define what has been measured and what should be measured - because what can be measured can be improved.^14^ You should consider collecting both quantitative and qualitative data because examining both types of data together often provides a more complete picture of educational continuity experiences compared to the use of a single approach.^15-17^

In the clinical setting, quantitative data can be collected from clinic schedules and converted using a continuity of care algorithm or tool, such as the Usual Provider Continuity (UPC) Index.^18,19^ A similar type of measure comparing learner attendance in clinic and health care provider schedules can be used to measure continuity of supervision. In the classroom setting, continuity of curriculum data can be retrieved from curriculum maps, class schedules, course content, and learning objectives from rotations.

While quantitative measurements are valuable and provide numbers that can be compared, they do not provide information on the quality of the educational continuity element being measured. Quality of experiences is as important – and sometimes may be more important than quantity of experiences.^20^ Qualitative data that provide information on participant perspectives on continuity experiences can be collected using surveys, individual interviews, focus groups or observation depending on the setting, the number of learners and teachers, and the resources available.

***3. Leverage electronic medical records (EMRs) as a tool for evaluating and improving educational continuity in the clinical setting***

Specific to educational continuity in the clinical setting, EMRs as both a data source for measures of educational continuity and tool for improving educational continuity is often overlooked. Continuity of care and continuity of supervision data can be collected using basic administrative functions of the EMR such as scheduling information to abstract patient/client demographics, visit dates, and learner and preceptor involvement. Continuity of curriculum can be measured through pulling EMR data about most common presentations encountered.

Barriers to using the EMR as a source of data include requirement of expertise in abstracting data and ease in interpreting the data obtained.^21,22^EMRs can vary in where data is entered and may not have fields such as who the learner is and the level of the learner. However, in the literature, there is research to show that it is possible to simply add a field such as identification of the learner in the visit and that such a field can improve not only data extractability, but also improve continuity of care by 25%.^23^

***4. Include patients and clients in evaluating and improving educational continuity experiences***

Another source of data about educational continuity is patients and clients. While patients and clients as a data source in classroom learning is very limited (although standardized patients can be a source of data in some circumstances), measures of continuity of care in the clinical setting can include data from and about patients. Even continuity of curriculum can be measured by talking to patients, especially those involved in programs such as patient partners.^24^ According to Towle et al.^25^ and McKeown et al.,^26^ “benefits to patients involved in education include satisfaction in giving back to the community, having an influence on the education of future professionals, and increased self-esteem and empowerment” (p. 20).^25^

***5. Don’t just identify barriers; seek solutions to improving educational continuity***

While the goal of all program evaluation projects should be to improve the existing program, one of the missteps in program evaluation in education is to identify a barrier and report it as a barrier without going further, or determine that the problem needs to be addressed through faculty development (source: 25% of the presentations at medical education research conferences).^27^ Carrying out program evaluation projects for educational continuity is an excellent opportunity to identify where barriers exist to implementation of educational continuity as well as a means to address or overcome those barriers. Arming learners, for example, with data about what is happening for them in educational continuity, as well as where barriers exist, can empower those learners to advocate for and make changes that would improve educational continuity.

## Summary

Educational continuity is a complex phenomenon. This complexity contributes to misunderstandings and irregularities in how it is implemented and especially how it is measured. However, it is possible to evaluate and improve educational continuity for our learners if we clearly identify and define what we are measuring when carrying out program evaluation. We hope that this article provides helpful tips on how to evaluate and improve educational continuity in health professions training.
